# Embedding and sustaining motivational interviewing in clinical environments: a concurrent iterative mixed methods study

**DOI:** 10.1186/s12909-019-1606-y

**Published:** 2019-05-22

**Authors:** David Lim, Adrian Schoo, Sharon Lawn, John Litt

**Affiliations:** 1 College of Medicine and Public Health, Flinders University, Bedford Park, Australia; 2 Centre for Remote Health: a Johanna Briggs Institute Affiliated Group, Flinders University, Alice Spring, Australia

**Keywords:** Motivational interviewing; lifestyle counselling; health behaviour change, Health education, Systems analysis, Implementation, Barriers, Fidelity

## Abstract

**Background:**

Motivational interviewing (MI) is internationally recognised as an effective intervention to facilitate health-related behaviour change; although, how it is best implemented and maintained in everyday clinical practice is not so clear. The aim of this study is to understand how MI as an intervention can be embedded and sustained in the clinical practice and learning environments.

**Methods:**

A concurrent iterative mixed methodology was utilised. Data collection occurred in two parts: a scoping review to identify reported barriers and enablers to embedding and sustaining MI in healthcare settings, and a survey of health professionals at an international clinical educator workshop on the topic. Results from both methods were integrated at the analysis phase (‘following a thread’) to understand how MI is embedded and the fidelity sustained in the clinical environments. Complexity theory as a conceptualising framework was utilised.

**Results:**

Eleven studies were included, and 30 health professionals were surveyed. Sustainability of MI at micro-clinical levels can be fostered through use of enabling technology, focus on patient-centred care, personnel development and process improvement. At the meso-organisational level, developing shared vision, creating opportunities and an organisational culture supportive of continuous learning are relevant issues. At the macro levels, adopting systems thinking and a learning organisation approach is important for sustaining MI.

**Conclusions:**

In addressing the recognised barriers to embedding and sustaining MI in health service provisions, clinical educators could potentially play a central role as change agents within and across the complex clinical system.

**Electronic supplementary material:**

The online version of this article (10.1186/s12909-019-1606-y) contains supplementary material, which is available to authorized users.

## Background

Motivational interviewing (MI) as a directive, patient-centred, collaborative counselling approach to activate and facilitate health behaviour change is internationally recognised as an effective intervention [1–5] and has been taught in health curricula for many years [6, 7]. However, there is evidence that MI skills are not always gained and retained. Furthermore, there is insufficient and often conflicting evidence that the common methods of training health professionals in MI (e.g. presentation followed by workshops that include practice) are sufficient to enable the development of competency in the method [8, 9]. There is ongoing discussion on the main components of MI and how they influence behaviour change [10–12]. This contributes to an ongoing discussion about the extent to which MI has been delivered as it was intended (fidelity) [10], and the nature and scope of training strategies that are both necessary and sufficient to lead to competency in MI and sustainability of its delivery [9] Many of the early findings show that many practitioners tend to revert to their old and less effective method of behaving/ counselling (i.e. telling people what to do without facilitating goal ownership) [13]. Whilst much has been written about the teaching of MI skills and enhancing outcomes for students [14], how MI is best implemented, maintained and supported in everyday clinical practice is less clear [9]. The types of behaviours for which MI is employed are highly variable, and MI as a clinical intervention is often delivered in combination with other medical and/or psychological interventions; therefore, the population, setting and context are of relevance [5]. It is possible that embedding and sustaining MI into routine clinical practice is one of those problems where any solution is likely to cause another problem or several problems (referred to by some as ‘wicked problems’ [15–17])^1^; nonetheless, it offers an opportunity to innovate and approach this matter differently. The aim of this study is to understand how MI as an intervention can be embedded and its fidelity sustained in complex clinical learning environments.

## Methods

A concurrent mixed methodology using a scoping review and survey was employed. The use of the two data sources was a pragmatic decision to better understand the complex social context in which health professionals learn about and then deliver MI. A scoping review was employed to examine macro/meso-system level influences and a survey of health professionals was conducted to collect micro-system level factors. The use of a micro-meso-macro framework had been used in other critical discourse analyses to understand and categorise social phenomena similar to that being studied here [18].

### Scoping review

We have anticipated heterogeneous studies in terms of *why*, *how*, *when*, by *whom*, *what*, and *where* MI would be used; for instance, the research purposes, the methods employed, duration of interventions, the participants involved (patients and providers, and sample size), the definition and measures of fidelity, the context and setting are relevant issues one would need to consider. Scoping reviews are “of particular use when a body of literature … exhibits a complex or heterogeneous nature … [and is] undertaken as exercises in and of themselves to summarize and disseminate research findings, to identify research gaps, and to make recommendations for future research” [19]. The non-discriminatory nature of this form of review permitted the inclusion of a broad range of factors that may affect and influence how MI can be embedded and sustained in a clinical learning environment and is reflective of the complex system in which clinicians and clinical educators operate. The Joanna Briggs Institute (JBI) methodology for scoping reviews was adopted [20]. In brief, the research question was identified, a three-step literature search of relevant studies were conducted, studies were selected using a team approach, data were charted and results collated to identify the implications for policy, practice and or research.

The Boolean search query included motivational interviewing [Mesh] AND (barrier OR challenge OR enabler OR facilitator) AND (implement OR maintain OR sustain OR embed OR integrate OR uptake OR adhere OR penetration) AND (fidelity OR effectiveness OR feasibility OR integrity OR safety OR quality OR strategy) and their respective truncated forms which were utilised for the broad literature search on PubMed, Scopus, Cochrane and JBI databases. The search was initially undertaken in March 2018, and subsequently updated in March 2019. All citations were imported into EndNote X8.2 (Clarivate Analytics, Philadelphia, PA, USA) for management. The inclusion criteria were: peer-reviewed; primary studies and systematic reviews; in English language; between 2008 – March 2019; the therapeutic intervention grounded in the MI principles in which the clinicians use rapport strategies to help the patient explore and resolve ambivalence about change [21]; in a clinical environment including but not limited to hospitals, primary healthcare, community healthcare centres and university teaching facilities; all geographical contexts. Exclusion criteria included: studies on MI itself; for example, psychometric properties, its efficacy or how to teach and assess it. Two reviewers (AS and DL) searched the databases independently and screened the citations by title and abstract. Reference lists of identified studies were further analysed for additional studies. Authors of primary studies were contacted for clarification if further information was required. Inclusion of selected studies was by consensus among all authors. A data extraction form derived from the JBI methodology for scoping reviews was used to capture the characteristics of the included studies, key information relevant to the research question, each study’s conclusions, implications for this research question, and weaknesses of each study [20]. The methodological quality of the included studies was assessed by at least two reviewers using the relevant JBI critical appraisal checklist. Any disagreement was resolved through discussion.

### Survey

An embedded quantitative and qualitative survey [22] conducted at an international clinical education conference workshop on MI held in May 2017 and attended by 30 health professionals was the primary source of data [23]. The aim of the international workshop was to explore how a tailored clinical micro-system could enhance practitioner competence in using MI as part of routine care, or interprofessional practice, to optimise health outcomes of patients with chronic conditions or who are at risk of developing these conditions. The workshop comprised of a written pre-workshop survey (quantitative data), focus group discussion (qualitative data), presentation of latest evidence and an outline of available resources, identifying possible barriers and enablers, and how clinical educators can develop a clinical microsystem to maintain best practice and outcomes for facilitating health behaviour change. Informed written consent was given by the conference organiser and participants prior to data collection. Human research ethics approval was granted by the Flinders University Social and Behavioural Research Ethics Committee. The written workshop questionnaire explored: (i) whether the participants use MI as a routine clinical intervention; (ii) their confidence in using it; (iii) how MI has been implemented; and, (iv) whether there are processes in place to support and monitor fidelity of MI delivery to patients. The questionnaire was developed a priori and the content validity was assessed through face validity and expert assessment – see Authors Information. The participants worked in groups to identify barriers to using MI at their workplace, the enablers and how MI can be maintained in a busy clinical environment. Responses to these areas of focus were captured qualitatively.

### Integration of data: following the thread

The results from the scoping review and survey were integrated iteratively at the analysis phase using the ‘following a thread’ methodology described elsewhere [24]. An initial analysis of each dataset was conducted to identify key themes and questions requiring further exploration. Specifically, the JBI meta-aggregative approach to the review of included literatures was conducted to identify key themes. The key theme linked to the purpose of this study, for instance, barriers to embedding MI in routine clinical environment (the ‘thread’) was followed through from the scoping review, and then through to the workshop with health professionals. This inductive-led framework for data cross-talk allowed for the initially qualitatively-framed question (e.g. barriers) to be elaborated quantitatively (e.g. frequency of use and self-rated confidence) to generate an overarching, multi-faceted understanding of how MI can be embedded and its fidelity sustained in the complex clinical practice and learning environment. All authors were involved in creating the initial themes from the scoping review, DL and AS were involved in survey data collection and in examining the data and links with the key themes, and SL and JL provided comments on themes. Group discussion finalised the threads.

## Results

### Scoping review

**Fig. 1:**
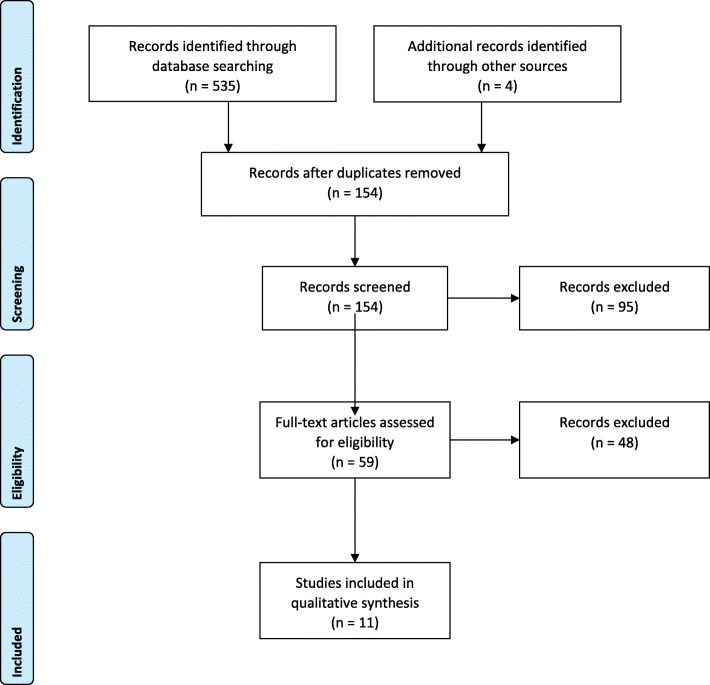
PRISMA flow diagram for the scoping review process

In the context of understanding how MI could be embedded or integrated and sustained in the clinical environment, Fig. 1 PRISMA flow diagram outlines the searching and screening process. Eleven papers were included in the scoping review. Key information from the included papers is summarised in Table 1. The studies took place in a variety of health service settings:

- acute settings [25],
- inpatients and residential settings [26],
- outpatient (intensive and regular) [26, 27],
- primary healthcare [28–31] and
- other community-based settings [26, 31–34]).

**Table 1:** Characteristics of included papers

Author	Type of study &amp; setting	Research aim	Intervention/methods	Results	Implications
Horn et al. (2008) [25]	Randomised controlled trial (*n* = 75), emergency department, USA	To determine the reach, implementation fidelity, and acceptability of a brief tobacco intervention for teens who had treatment in a hospital emergency department	A 15–30 min motivational interviewing session together with an educational handout, a postcard after the visit and 3 follow-up phone calls (1,3 and 6 months)	Low levels of reach, high levels of implementation fidelity, and high levels of acceptability for teen patients, their parents, and emergency department personnel.	It is possible to implement a motivational tobacco intervention that includes an interview session in an acute, busy and unpredictable setting. Enabler: structure, time, resource, staff buy-in
Jansink et al. (2010) [28]	Qualitative interview (*n* = 12), nurses in general practice, the Netherlands	To examine barriers nurses encounter in lifestyle counselling to patients with type 2 diabetes to inform the development of an implementation strategy to improve lifestyle behaviour change in general practice	Broad questions on perceived barriers encountered by nurses at three levels (nurse, patient, practice) on management of type 2 diabetes	Nurses reported lack of counselling skills and insufficient time as barriers in effective lifestyle counselling (since patients had limited knowledge of a healthy lifestyle and insight into own behaviour, and lacked motivation to modify lifestyles or the discipline to maintain an improved lifestyle).	An implementation strategy based on motivational interviewing can help to overcome ‘jumping ahead of the patient’ and promotes skills in lifestyle behavioural change. Agenda setting and prioritizing the behaviour change will assist to develop social maps that contain information on local exercise programs. Barrier at patient level: knowledge, attitude, skills and compliance. Barrier at practice level: organisation processes, staff, capacity, resources and structure
Amodeo et al. (2011) [26]	Phone interview (*n* = 172), providers in community-based addiction treatment organisations, USA	To explore barriers to implementing evidence-based practices (including motivational interviewing) in community-based addiction treatment organisations	Comparing staff descriptions of barriers for motivational interviewing, adolescent community reinforcement approach, assertive community treatment, and cognitive-behavioural therapy	Staff described different types of barriers. For motivational interviewing, the majority of barriers involved insufficient training, variance in training and perspective, staff resistance, client resistance and organizational factors such as philosophy, time, space, process and structure	Need to include explicit strategies to address such barriers, and consider whether their programs have the organisational capacity and community capacity to meet the demands
van Eijk-Hustings et al. (2011) [30]	Concurrent mixed methods, primary and secondary care setting, the Netherlands	To examine the uptake of motivational interviewing in daily practice by health care professionals in a care management initiative for patients with diabetes in the region of Maastricht, the Netherlands	Directly and six months after the staff training (n = 2 practice nurses, 4 diabetes specialist nurses, 4 dieticians) the application of motivational interviewing was measured objectively using the Motivational Interviewing Treatment Integrity Scale coupled with patient clinical data (*n* = 91 intervention vs. 50 control). Interviews with the 10 trained and another 10 untrained professionals were conducted to understand barriers and enablers	The applicability of motivational interviewing in daily practice was found feasible, with various degrees of uptake. Mostly uncomplicated techniques were applied. Professionals stated the need for training and practice to be able to apply more complicated techniques	In daily practice, a phased training in motivational interviewing is recommended, with sufficient time and support by colleagues as essential conditions to profit most from the training sessions
Rosseel et al. (2011) [29]	Embedded interviews (*n* = 62) in a controlled study, primary dental care centre, the Netherlands	To encourage primary care dental professionals to use a stage-based motivational protocol to provide more smoking cessation advice and support for all smoking patients in the Netherlands	A smoking cessation protocol was introduced in 23 primary care dental practices in the Netherlands in 2008. Practices could choose between a minimal or optimal version of the protocol, including motivational interviewing training. Patients were asked whether they had received smoking cessation advice and support as part of their treatment.	Lack of practice time and anticipated resistance on the part of the patient were cited as barriers by over 50% of the dental professionals in the first interviews. Periodontal treatment and the presence of smoking-related diseases were mentioned as the most important stimuli	Education on the associations between smoking and oral health, vocational training on motivational interviewing and the offering of structured advice protocols were identified as promising components for an implementation strategy to promote the involvement of dental professionals in the primary and secondary prevention of tobacco addiction
Lundgren et al. (2011) [33]	Phone interviews (*n* = 100 program directors), community-based addiction treatment organisations, USA	To explore implementation of evidence-based practices including motivational interviewing in community-based addiction treatment organisations	Describes community based addiction program director attitudes on: (1) satisfaction with program they were mandated to implement; (2) the extent to which their organisation modified the program; (3) reasons for modifications; and, (4) the standards they used for modifications	Program directors were highly positive about program implemented and modifications made. Most common modifications were adding or deleting intervention sessions to serve the needs of a specific client population	Government funders require community based addiction treatment organisations to implement and maintains standards. Conflict between providing evidence-based practices and culturally appropriate services
Rosseel et al. (2012) [35]	Review (*n* = 8), smoking cessation in dental care	To summarise evidence regarding the effectiveness of various implementation strategies to stimulate the delivery of smoking cessation advice and support during daily dental care	Search of online medical and psychological databases, correspondence with authors and checking of reference lists. Eight studies or were included with four deemed to be at sufficient quality	Professional education may enhance motivation for smoking cessation activities and advice giving together with organisational interventions (e.g., protocols, involvement of the whole team, referral possibilities) and incorporation of patient-oriented tools	Multifaceted support strategies positively influence dental professionals’ knowledge of smoking and smoking cessation, their motivation to give advice and their performance
Lundgren et al. (2012) [34]	Phone interviews and web survey of staffs working in community-based addiction treatment program, USA	To examine the relationship between clinical staff (*n* = 510) and director (*n* = 296) perceptions of organisational capacity and lever of barriers experienced when implementing new intervention	Organisational readiness for change and phone interview to understand barriers	Barriers: stress, low level of program needs, working in a program that had been in existence for a short period,	Staff who implemented motivational interviewing techniques as compared to other interventions and in program in existence for short period experienced lower level of barriers
Lundgren et al. (2013) [32]	Phone interviews and web survey, staffs working in community-based addiction treatment program, USA	To explore whether staff perceptions about the organisational capacity of their treatment unit are associated with staff experience of barriers to implementing evidence-based addiction treatment practices	Prior studies have identified that working in an addiction treatment unit with higher levels of organisational capacity is a factor associated with positive staff attitudes	Barriers identified from bivariate analysis: clinical staff who had five or more years of addiction counselling experience and less frequently implemented new counselling interventions, staff who reported a less level of influence in the organisation	Government funders of community addiction programs must take organisational capacity into account, continued funding is needed to promote adoption and adherence, ongoing training opportunities to promote implementation
Guerrero and Kim (2013) [27]	Multi-methods evaluation, USA	To explore the extent to which pressure from funding and regulation, leadership and readiness for change impact on organisational implementation	Online survey, review of program, qualitative interviews and review of printed material available at 122 addiction programs	Public funding and regulation associated with greater implementation; leadership capacity associated with outreach to minority and development of diverse staff; programs with more graduate staff were associated with less involvement from communities	Investment in funding, leadership skills and strategic climate are importance enablers
Aboueid et al. (2019) [31]	Qualitative interviews (*n* = 14), dietitians, Canada	Clinicians’ perspectives on barriers and enablers	Naturalistic inquiry approach of 14 dietitians	Individual levels enablers: financial resources, education, self awareness. Relationship-level enablers: supportive networks. Community-level enablers: community program, workplace norm, supportive interdisciplinary teams.	Macro system barrier such as socioeconomic status, discrimination, lack of communication between providers can impede sustainability.

Abbreviation: *USA* = United States of America

The included studies were conducted in USA [25–27, 32–34], Canada [31] and Netherlands [28–30]; and with a variety of health professionals:

- nurses [25, 28, 30],
- physician assistants [25],
- dentists [29] and dental hygienists [29],
- dieticians [30, 31],
- social workers [25],
- counsellors [27, 32],
- psychologists [25],
- public health practitioners [25] and
- program directors and clinical supervisors [27, 32–34]).

The type of interventions varied and included: smoking cessation [25, 29, 35], diabetes management [28, 30], weight management [31] and addiction treatment [26, 27, 32–34]. A number of system barriers and enablers to implementing and maintaining MI in these health services were identified and presented in Table 1.

In the community-based addiction treatment setting where MI was predominately used for the prevention of lifestyle-related behaviour that impacted on health (e.g., smoking, physical activity, hazardous drinking, dietary patterns), Lundgren and colleagues found through a large national study [26, 32–34] the following barriers at the microsystem levels: staff and client resistance to change; the skills and confident of practitioners; and perceived needs and ability to influence/ change behaviour. At the meso-organisation levels, the study identified the importance of explicit strategic vision for incorporating MI in routine health service provision; the willingness and facility to foster adoption of MI through ongoing staff development and effective change management; enabling administrative processes and positive organisational leadership. The study also identified the importance of being able to tailor programs and services to suit appropriate sociocultural needs so as to address community demands. It was argued that performance (i.e., evidence-based practice and cost-effectiveness) can be linked to funding as an enabler for organisations and their health professionals to facilitate the adoption of MI. Similar findings were reported in a separate USA study of addiction health services by Guerrero and Kim [27]. In this study, the authors found positive correlation between funding and greater implementation (β = 0.20, SE **=** 0.90, *p* &lt; 0.05) and enabling policies and procedures (β = 0.19, SE **=** 0.90, *p* &lt; 0.05).

In the area of dentistry where MI is used in both the modification of unhealthy behaviour and promotion of healthy oral health behaviour, two studies [29, 35] identified the following micro-clinical level enablers:

- Client factors: choice and motivation, presence of consequence of the health-related behaviour such as smoking-related disease;
- Clinicians’ factors: training, availability of patient-orientated resources; and
- Clinical team factors: involvement of the whole team, ability to refer out, involvement of other providers, availability of other providers, measure of clinical team performance, and clear protocol and procedures.

Horn et al. utilised MI for smoking cessation in an acute care setting and reported the additional factors of staff buy-in and the integration of MI intervention into routine clinical work at the micro-level as enablers [25].

In the Canadian study on weight management, supportive networks and interdisciplinary teams were found to be meso-level enablers in sustaining MI [31].

Nurses in one diabetes care study perceived that most of the barriers were at the level of the patient due to them showing limited knowledge of what a healthy lifestyle is, having poor insight into their own behaviour as a risk factor, and lacking motivation and discipline to enhance their lifestyle and maintenance of positive lifestyle [28]. This is a common finding akin to blaming the victim and suggesting that their preferred strategy is to tell people what to do rather than tap into the patient’s expertise and suggested strategies as a facilitator. Furthermore, in this study the nurses also perceived that they lacked the needed MI skills and that they had insufficient clinical time to be effective in lifestyle counselling. By comparison, in a separate diabetes care study, the clinicians reported that MI as part of daily practice is feasible especially the use of less complicated MI techniques [30].

### Survey

Most workshop participants (93%) reported infrequent (‘not often’ or ‘sometimes’) routine use of MI in current clinical practice and low degree of confidence in using MI (median 1.5 out of 10 [0 = not confident, 10 = very confident], IQR 2.5; see Additional file 1), despite the majority of participants being senior clinicians and or clinical educators (87%; *n* = 26). Assessment of the fidelity of the MI intervention was often conducted, 54% of the participants reporting some form of monitoring. The types of outcome measures assessed were dependent on the context, for instance, in respiratory medicine and smoking cessation, “*monitored days without a smoke or cigarettes*”; and in drug and alcohol, “*regular follow-up on* [adherence or compliance with] *mandatory treatment*” were reported.

The workshop participants identified that “*time is a constraint*” in routine implementation of MI in busy clinical and teaching practice, and the participants felt that dedicated clinics or time needed to be “*set aside solely for* [practising] *MI*”. The participants identified “*working in a tertiary hospital*” as a barrier to successfully adopting the use of MI in routine clinical practice due to the lack of infrastructure support and buy-in from administration. Specifically, junior participants (*n* = 4; interns) reported that MI was “*not really relevant to my current practice*” due to inadequate role modelling and continuous professional development opportunities, and “*having just come out of residency training, the ways we were monitored is still fresh in my mind. We had an OSCE* [objective structured clinical examination] *for this. However, things are different out of residency. There is no time to do motivational interviewing or someone to supervise me*”.

Reported facilitators for monitoring fidelity included process enablers: i.e. recalls, the use and availability of “*multidisciplinary team (e.g. diabetes educator)*”, and embedding MI as part of routine practice “*I think the most important component is small amounts of motivational interviewing over multiple consults*”.

### Thread: embedding and sustaining motivational interviewing

Health care has been described in the literature as a non-linear, complex adaptive system due to the multiplicity of actors, agents, systems and controls; different values, perspectives and needs; competing priorities and ideology; combination of planned and emergent change; being more effective and transformative at the expense of increased costs and greater potential for harm. There is an increased recognition that systems thinking is necessary to tackle complex health problems due to the dynamic interplay between biological, social, physical, cultural and economic factors. The many stakeholders and issues involved make embedding MI in healthcare practice complex because MI practice and processes involve micro-, meso-, and macro-system levels with fuzzy boundaries between them and nested within one another. Therefore, using complexity theory, systems thinking could assist in conceptualising a framework that can be individualised to best respond to local and regional contexts to facilitate sustainability of MI practices. Complexity science is “the study of the dynamics, conditions, and consequences of interactions within a complex system” [36] that, in turn, is nested within other systems, where complex occurrences are varied and self-organisation takes place to construct some order. Uncertainty and ambiguity are features of complex systems, whilst disagreement and other problems are not regarded as obstacles but rather as opportunities for change [37]. As such, complex systems provide learning opportunities and transformation through reflection and self-organisation [38] that may lead to innovation and change.

At the healthcare clinical microsystem level, the MI practitioners provide direct care to the patients or clients (therapeutic) and liaise with other providers (working alliance), while at the same time in their dual role as clinical educator/ supervisor/ preceptor they offer an empathetic and supportive mentorship to emerging and junior clinicians.

This micro-system is the building block of the organisation where there are common clinical and/or business aims, information shared, processes linked, services rendered, and performance outcomes measured. At this adaptive system level, the sustainability of MI can be fostered through: organisational support and positive leadership for a shared purpose and goals; adopting system thinking and use of enabling technology and a supportive environment for team learning and personal mastery; and focusing on patient-centred care, personnel development, interdependence of health care team, process improvement, and reasonable output and performance measures.

The mediating mesosystem level may take different forms depending on the context and purpose. In the integration and incorporation of MI within therapeutic health care delivery, this may take the form of multidisciplinary, interprofessional or transprofessional practice; acute versus chronic condition management; planned or unplanned episodic and longitudinal care; as well as funding, partnership and interaction with other agents; enabling processes and policies to integrate MI into routine health care service provision. In clinical education, the teaching, attainment and maintenance of MI competency may have its lens on the curricula (horizontal, vertical, hidden), pedagogy, infrastructure and environment, course and professional accreditation and standards to attain. While in implementing and sustaining MI in the health service management may require leveraging different microsystems and funding sources, creating opportunities and support, developing shared vision and fostering congruent organisational culture, and being responsive to local and regional health needs.

Embedding MI within the broader socio-politico-economic macro-system levels would require policy and decision makers to recognise system-thinking perspectives and a learning organisation approach in their deliberations, and develop bipartisan strategies that are long-term, span jurisdictions and sustainable. Implementing and maintaining MI within health care can be problematic; for instance, in the way health professionals are currently educated, in the presence of professional silos and prevailing culture, scope of practice, and due to the nature of remuneration and funding (e.g. fees for service as compared to fees for performance). Therefore addressing one aspect of such a problem in a single system may create other challenges and problems within and across other systems.

## Discussion

Successfully embedding MI in routine patient-centred health care requires awareness of the possible barriers, and facilitating agreed guidelines and processes that not only support education, skills development and maintenance of MI, but also allow time and provide support for interprofessional collaborative practice at the departmental or micro-level. As such, and from the perspective of a learning organisation [39, 40], clinical educators could play a central role as change agents within and across this complex systems to support evidence-based clinical practice that includes MI through personal mastery, teamwork, mental models and vision. Personal mastery refers to enhancing capability of the individual clinical educator and the clinical team (including students) to apply MI in a specific context/ setting. Teamwork takes place predominantly in the clinical microsystem level. Mental models (personal beliefs and attitudes) relate to all three systems (micro, meso and macro); whereas vision is much broader and has more an external focus (i.e., what does the organisation stand for, and how do personal actions support this?). Tensions within and between the systems, dimensions and domains can make it challenging to create a work environment and culture that optimises the routine use of MI, particularly in the prevention and management of chronic disease. For instance, the micro-level can include patient and staff resistance, and high clinical workloads with opposing education and training demands [25, 26, 28, 29, 32–35]. At the meso-level, issues can include poor research culture and organisational support for mentoring, peer reinforcement and educational innovation, linear (bureaucratic) command chain and rigour of practice together with limited access to continuing professional development [26, 28–30, 32, 41]. At the macro-level, shifting socio-political priorities can make it difficult to successfully embed MI at the coalface of health service delivery. Therefore, to be an active participant and agent for change, the clinical educator needs to navigate through these challenges to meet personal, educational, health services, professional and societal expectations. This requires investment in the necessary mean and mode for effective communication to facilitate exchange of information and sharing of best practice using enabling technology and explicit *modus operandi* [23]. Overcoming MI implementation challenges also requires strategic leadership which fosters an empowering workplace culture that promotes inquiry and dialogue, encourages cross-systems connection, collaboration and team learning, and shared vision [42]. It also requires a transformative organisational culture that permits challenges to conventional practices and assumptions. This may include interprofessional collaborative practice at the micro-level, with individual competency and monitoring of performance, and actively facilitating departmental processes that support MI practices. Meso- and macro-level related factors to be considered include local and regional health needs, distinguishing between acute versus chronic health management (urgent versus important – i.e. something that is urgent is not necessarily important), competing socio-political priorities and associated funding. Recognising the different stakeholders and their interdependence, engaging them and working towards joint ownership, is likely to result in more effective and sustainable models of care that serve regional/ local communities and their members. Additional benefits may include improved health career pathways, and greater funding opportunities and job satisfaction.

### Possible roles of clinical educators

Successful implementation and integration of MI within clinical practice, and maintaining fidelity, depend on factors such as training and associated supervision/coaching, levels of motivation or resistance of the client or counsellor, departmental and organisational support, and process-related issues. Clinical educators’ roles in health service agencies are therefore not limited to education and training, but also includes leadership [40]:

1. Education and training of MI:
  Supporting staff and their students on placement
  Providing opportunities for staff and students to practice MI in the clinical setting
  Ongoing supervision and coaching of MI in the clinical environment.
2. Leadership:
  Embedding MI within client-centred health care requires awareness of the possible barriers and facilitating agreed guidelines and processes
  Micro level: facilitating departmental processes that support MI
  Meso level: facilitating justified organisational support
  Macro level: liaising with universities (e.g. curriculum reform if or as needed), professional groups to best support clinical practice in line with organisational capacity and response to community needs, professional standards and associated training requirements, local and regional health needs, acute versus chronic condition management, and associated funding.

### Performance-based enablers

Performance-based enablers have been recognised as another possible mechanism to the development, implementation and sustainability of MI in health service provision to change health behaviour in people at risk, and to evaluate its impact and cost-effectiveness [27, 30, 32]. These enablers are located on the interplay between the macro/ meso and the micro system levels. More specifically:

- Performance indicators such as those associated with effectiveness, safety and access as imposed by governance (macro level) need to be realistic and facilitate enabling processes and associated outcomes on the organisational and department levels (meso and micro levels), with a focus on impact and sustainability (focus on the longer term)
- There should be adequate funding and duration of funding to develop capacity, as well as flexibility to evolve so as to be adaptive and responsive to the relevant needs in the community
- Encourage collaboration, partnership and benchmarking between agencies – value for money and synergism
- Performance indicators must be transparent, accountable and responsive
- Adoption of clinical guidelines that are based on best practice and endorsed by the relevant professional bodies
- The consideration of the minimum ‘dose’ of MI (on the departmental/micro level) to be effective, intervention may include longitudinal planned follow-up
- The need for ongoing professional development
- Robust governance process.

### Policy enablers

Between macro and meso levels, policy is an official statement of intent by the organisation to guide decision-making and service delivery on the micro level. Enabling policy could ensure consistency of approach; enable shared communication and understanding; efficient and effective co-ordination of health service provision such as necessary time, resources, training and remuneration for the integration of MI in its routine business case. Policy enablers could influence staff expectations and performance to meet the required performance indicators and health needs of the communities [26–28, 31, 32]. Issues to be considered include:

- Development in consultation, based on sound evidence and a realistic possibility of being contextualised
- Room for individualisation whilst providing a congruent approach that has bipartisan support
- Micro level: provides clarity, common language/ goals
- Meso level: articulate inwards and outwards the organisation’s visions and values
- Macro level: system thinking approach, not prescriptive but outlined principles and goals to facilitate enabling processes
- Planned schedule of revision to harness when new evidence and technology become available, and/or when community expectation and priority shifted.

### Recommendations

- Policy makers, designers and funders of interventions (such as MI) could include clear strategies and develop standards to address the possible barriers to implementation and sustainability of those interventions, and ensuring maintenance of fidelity in their use in practice.
- Health service programs proposing to use MI must consider explicitly the community acceptance and needs as well as the organisational capacity to make the service sustainable.
- Phased training in MI is preferred, with sufficient time between sessions and with support by peers.
- The aim should be creating capability in MI practice rather than competence so that professionals are prepared for new and evolving situations.
- Adopting the principles of a learning organisation through a shared vision, values and goals, and empowering people at the clinical coalface by reframing challenges and problems as learning opportunities.
- Clinical educators are likely to be well-suited to implement and maintain evidence-based practice in complex systems to facilitate behavioural change.

## Conclusion

Although MI training is a starting point, evidence indicates that organisational systems that support processes such as mentoring, supervision, and peer reinforcement are all needed as ongoing features. Results of this study indicate that inadequate system provisions are a key barrier to ensuring MI is routinely embedded within clinical environments. Successful implementation and integration of MI within clinical practice and maintaining fidelity depends on factors such as training, motivation, departmental and organisational support for MI (including workload), and process enablers. Possible roles for clinical educators in sustaining MI in micro-clinical learning environments include: facilitating departmental processes that embed MI as routine evidence-based clinical practice; facilitating organisation justification and support at the meso-level; and, liaising with universities and professional groups to best support clinical practice in response to community needs at the macro-level.

## Additional file

Additional file 1: Summary of survey findings. (DOCX 23 kb)

## Abbreviations

JBI: Joanna Briggs Institute
MI: Motivational Interviewing
USA: United States of America

## Footnotes

1 A ‘wicked problem’ is one that requires reassessment of some of the traditional way of working and solving problems due to the interrelationships among the full range of causal factors underlying them. Each attempt to create a solution changes the understanding of the problem. Wicked problems cannot be solved in a traditional linear fashion, because the problem evolves as new possible solutions are considered or implemented. It therefore requires broader, more collaborative and innovative approaches.
