# Supplementary material for: Embedding and sustaining motivational interviewing in clinical environments: a concurrent iterative mixed methods study
Source: BMC Med Educ. 2019 May 22;19:164. doi: 10.1186/s12909-019-1606-y (PMC6529989; doi:10.1186/s12909-019-1606-y)
Supplement: Supplementary file 1 — Summary of survey findings. (DOCX 23 kb) [file 12909_2019_1606_MOESM1_ESM.docx]

Additional File 1: Summary of survey findings

| 1. How often do you use motivational interviewing in your current practice? | | | | | | | |
| --- | --- | --- | --- | --- | --- | --- | --- |
| Never  0 % | Not often  40 % | | Sometimes  53 % | | Mostly  0 % | | Always  7 % |
| 1. How confident are you in using motivational interviewing? | | | | median 1.5 out of 10, IQR 2.5  [0 = not confident, 10 = very confident] | | | |
| 1. How does your practice implement motivational interviewing? | | | | | | | |
| - *We don’t* - *No time to do motivational interviewing* - *Smoking cessation visits* (n=2) - *Data tools like PenCAT* - *Clinical placement* - *Open ended questions* (n=2) - *Introduced during care planning with chronic disease* - *Relationship building with patient* | | | | | | | |
| (iv) Does your practice have processes in place to support and monitor the fidelity of motivational interviewing? | | | | | | | |
| Not at all  0 % | | Not really  46 % | | Yes, somewhat  39 % | | Yes, mostly  15 % | |
